# Supplementary material for: Overexpression of the X-Linked Inhibitor of Apoptosis Protein (XIAP) in Neurons Improves Cell Survival and the Functional Outcome after Traumatic Spinal Cord Injury
Source: Int J Mol Sci. 2023 Feb 1;24(3):2791. doi: 10.3390/ijms24032791 (PMC9917926; doi:10.3390/ijms24032791)
Supplement: Supplementary file 1 [file ijms-24-02791-s001.zip › ijms-2099818-supplementary.pdf]

Supplementary Table S1

Cell Line	Reference
SH-SY5Y human neuroblastoma	ATCC Cat#CRL-2266<br>RRID:CVCL_0019
**Immunoblot Primary Antibodies**	**Reference**
α-c-Myc	Roche Cat#11667149001<br>RRID:AB_390912
α-hXIAP	BD Biosciences cat#610717<br>RRID: AB_398040
α-Caspase3	Cell Signaling Technology cat#9662,<br>RRID: AB_331439
α-Caspase 7	Cell Signaling Technology cat#9492,<br>RRID: AB_2228313
α-Poly (ADP-Ribose) Polymerase (PARP)	Cell Signaling Technology cat#9542,<br>RRID: AB_2160739
α-β-Actin	BD Biosciences cat#612656<br>RRID: AB_2289199
α-β-Tubulin	Sigma-Aldrich Cat# T5293,<br>RRID:AB_477580
**Immunoblot Secondary Antibodies**	**Reference**
HRP-conjugated goat α-Rabbit	ThermoFisher Scientific cat#31460;<br>RRID: AB_228341
HRP-conjugated goat α-Mouse	ThermoFisher Scientific cat#31430;<br>RRID: AB_228307
**Immufluorescence Antibodies**	**Reference**
Rabbit-α-NeuN	ThermoFisher Scientific cat#ABN78;<br>RRID: AB_10807945
Alexafluor 488-conjugated goat α-Rabbit	Abcam, cat#150081;<br>RRID: AB_2734747
**PCR primers**	**Sequence**
hXIAP Primer forward	**5'-CCCAAATTCAACAAACCG-3'**
hXIAP Primer reverse	**5'-CTGAACTGAGTAGGACGT-3'**
